# Scalpel can achieve better clinical outcomes compared with electric cautery in primary total knee arthroplasty: a comparison study

**DOI:** 10.1186/s12891-020-03457-1

**Published:** 2020-06-29

**Authors:** Wei Lin, Yike Dai, Jinghui Niu, Guangmin Yang, Ming Li, Fei Wang

**Affiliations:** Department of Orthopedic Surgery, Third Hospital of Hebei Medical University, No. 139 Ziqiang Road, Shijiazhuang, 050051 Hebei People’s Republic of China

**Keywords:** Scalpel, Electric cautery, Forgotten joint score, Total knee arthroplasty

## Abstract

**Background:**

Whether using the scalpel can provide better and faster recovery after the primary total knee arthroplasty (TKA) is still controversial. The aim of this research was to compare the clinical outcomes of using the scalpel and the electric cautery in primary TKA.

**Methods:**

From January 2016 to December 2017, a retrospective cohort study was conducted in 313 patients who underwent unilateral primary TKA by using the scalpel (group S). During this period, we selected 313 patients who underwent unilateral primary TKA by using the electric cautery (group E) for comparison. The tourniquet time, operative time, blood loss, wound complications, visual analog score for pain, range of motion, Knee Society Score were assessed between the two groups. The Forgotten Joint Score was used to analyze the ability to forget the joint.

**Results:**

There were no significant differences in tourniquet time, operative time, blood loss between the two groups (*p* > 0.05). In the group S, the visual analog score for pain, range of motion, Knee Society Score were found better results at 1 week, 1 month, 3 months, 6 months, 12 months and 24 months after surgery (*p* < 0.05). Besides, during the follow-up period, the Forgotten Joint Score was significantly higher compared with group E (*p* < 0.05).

**Conclusion:**

In this research, the patients who underwent TKA by using the scalpel could achieve better clinical outcomes. In addition, if forgotten artificial joint after TKA was the final goal, the patients who underwent TKA by using the scalpel would acquire better quality of life.

## Background

More than 100 million people around the world are affected by osteoarthritis (OA) [1]. It is a chronic disease, the knee joint is the most severely affected joint, and there is no doubt that total knee arthroplasty (TKA) is the most effective surgical method for the treatment of end-stage knee OA [2]. The main advantages of TKA are to relieve the pain of the knee, improve the knee function, restore lower limb alignment, and improve the quality of life of patients [3]. In the process of TKA, some surgeons like to use the scalpel and others like to use the electric cautery to expose the articular cavity. However, which of these two methods is better is still controversial.

In the past 10 years, many surgeons believe that using the electric cautery can reduce blood loss during surgery, so there is a tendency to use electric cautery instead of the scalpel [4, 5]. But many experienced orthopaedic surgeons still prefer to use the scalpel to cut skin and soft tissue quickly and efficiently to complete the TKA [6]. The electric cautery is one of the conventional instruments in surgery, and it has the advantages of good hemostatic effect, convenient and simple operation, etc. The unipolar electric cautery is a traditional device that transfers current through a resistance wire electrode to generate heat, which is used for tissue coagulation [7]. Although using the electric cautery in surgery is very effective, it has the potential to cause complications. At the end of the twentieth century, the incidence of electrosurgical injuries remained at about 2 to 5 per 1000 people [8, 9]. One study discovered that using electric cautery during surgery resulted in poor wound healing and high wound infection rates [10]. Some studies have noted that using the electric cautery during surgery not only increases the cost of the operation, but also generates a lot of harmful smoke during the operation, which affects the health of the surgical staff [11, 12]. Because the electric cautery generates a lot of heat, mistakes in operation may lead to more soft tissue damage [13, 14].

We found few studies comparing the clinical outcomes in primary TKA by using the electric cautery or the scalpel. Consequently, we conducted a retrospective, and case matched research to compare the clinical outcomes between the two methods, with a follow-up of at least 2 years. We assumed that using the scalpel might achieve better clinical outcomes compared to the electric cautery in primary TKA.

## Methods

With the approval of the Institutional Review Committee, we performed a retrospective case-matched study from January 2016 to December 2017. Three hundred thirteen patients who underwent unilateral primary TKA by using the scalpel from January 2016 to December 2016 were included in group S. From January 2017 to December 2017, we selected 313 patients who underwent unilateral primary TKA by using the electric cautery (group E) for comparison. To improve the reliability of this research, the two groups matched in a 1:1 ratio based on age, sex, body mass index (BMI), and follow-up time. Inclusion criteria were (1) unilateral primary knee osteoarthritis; (2) flexion-contracture deformity < 20°; (3) varus deformity < 20°. Patients who had valgus or stiff knee, neurological problems, anticoagulant therapy, revision TKA, inflammatory arthritis, or previous open knee surgery were excluded. All surgeries were performed in our center by the same senior orthopaedic surgeon.

### Surgical technique

In the two groups, the skin was incised with a scalpel, 10 mg/kg dose of tranexamic acid (TXA) was administered intravenously to all patients before surgery, and after the implant was placed, 2.0 g TXA mixed with 100 ml of normal saline directly into the surgical site and soaked in the solution for 2 min [15]. According to the patient’s physical condition, using hypotensive anesthesia with low mean arterial pressures. All surgeries were accomplished through the medial parapatellar arthrotomy, and pneumatic tourniquets were used during surgeries to help stop bleeding. In the group S, we used scalpel number 22 (Jinhuan® surgical blade, Jinhuan medical supplies Co., Ltd., Shanghai Pudong, China) for the midline skin incision, and then scalpel number 10 (Jinhuan® surgical blade, Jinhuan medical supplies Co., Ltd., Shanghai Pudong, China) to dissect the sub-fascial layers and opened the joint cavity by the incision of the medial retinacular tissue. Then release the medial deep collateral ligament. The synovium, meniscus and anterior cruciate ligament were removed. Some major small blood vessels around the knee joint did not coagulate, and the tourniquet was released when the wound was closed. In group E, using the same number scalpel for the midline skin incision. Then, using electric cautery (Peng’s Multifunctional Operational Dissectors (PMOD), Co., Ltd., Zhengjiang Shuyou, China), set at 65 W coagulation mode, to separate and cut the soft tissues in the knee joint cavity mentioned above. After the implant was placed, the tourniquet was released, and the bleeding points were coagulated by the electric cautery during the surgery. All patients received the same type of knee prosthesis (CR, Mobile bearing, LINK, Germany, Gemini MK II) and postoperative treatment options, including rehabilitation programs and pain control.

### Outcome evaluation

The assessments were conducted by a senior orthopaedic surgeon who did not participate in the treatment. Patients demographics in regard to age, sex, BMI, follow-up time, operative time, tourniquet time, blood loss, wound complications were examined. Routine blood tests were performed 1 day before and 1 day after surgery to avoid the effect of perioperative infusion on the test results.

The range of motion (ROM), visual analog score (VAS) for pain, Knee Society Score (KSS) [16] were assessed. For comparing the postoperative status of the patients who received TKA via the two different methods, we used the Forgotten Joint Score (FJS; a 12-item questionnaire with a maximum of 100) to analyze the ability to forget the joint [17]. Higher scores represented better results. All data were assessed at 1 week, 1 month, 3 months, 6 months, 12 months, and 24 months after surgery.

### Statistical analysis

All statistical data were used the Shapiro-Wilks test to check the normality of continuous variables. If the data were normally distributed, the two groups would be compared using t-test; if not, the non-parametric test would be performed. And the Fisher’s exact test was performed for categorical variables. The correlation between the FJS score and surgery method (scalpel versus electric cautery), sex, gender and BMI were analyzed by multiple linear regression. The data were analyzed with the SPSS 19.0 (IBM, Chicago, Illinois, USA). *p* < 0.05 was considered statistically significant.

## Results

All patients in both groups were followed up at least 2 years. No significant differences were found between the two groups in regard to age, sex, BMI, ROM, VAS, KSS, operative time, blood loss, tourniquet time before surgery (*p* > 0.05) (Tables 1, 2). Wound complications accounted for 2 cases (0.6%) in group S; 9 cases (2.9%) in group E (*p* = 0.033). No revision surgery was performed in either group.

**Table 1:** Patients demographics in the two groups

Demographics	Group S	Group E	*p*-value
Total patients	313	313	–
Age (years)	68.1 ± 4.2	67.3 ± 5.2	0.532
BMI (kg/m^2^)	26.7 ± 4.2	27.4 ± 3.2	0.491
Sex			0.845
Male	66 (21.1%)	68 (21.7%)	–
Female	247 (78.9%)	245 (78.3%)	–
Side (right/left)	170/143	162/151	0.442
Follow-up time (years)	2.1 ± 0.3	2.2 ± 0.4	0.661

*BMI* Body mass index; mean ± standard deviation

**Table 2:** Postoperative clinical results in the two groups

Results	Group S	Group E	*p*-value
Operative time (min)	82.9 ± 10.1	81.6 ± 10.4	0.457
Tourniquet time (min)	38.1 ± 3.4	37.8 ± 3.6	0.341
Pre-operative haemoglobin (g/dL)	12.4 ± 1.2	12.1 ± 1.6	0.281
Post-operative haemoglobin (g/dL)	10.3 ± 1.1	10.2 ± 1.6	0.128
Pre-operative hematocrit (%)	42.1	43.3	0.361
Post-operative hematocrit	36.2	35.8	0.413
wound complications	2 (0.6%)	9 (2.9%)	0.033

mean ± standard deviation

In the group S, the results of VAS, ROM, and KSS were better than those in the group E at 1 week, 1 month, 3 months, 6 months, 12 months and 24 months after surgery (*p* < 0.05) (Tables 3, 4). Furthermore, during follow-up, the FJS score in group S was significantly higher than that in group E (*p* < 0.05) (Table 5). The multiple linear regression showed that higher FJS score was correlated with the scalpel method (*p* < 0.05) (Table 6).

**Table 3:** The VAS and ROM in the two groups

Results	Group S	Group E	*p*-value
VAS			
Preop	5.1 ± 1.4	4.9 ± 1.6	0.351
Postop 1 week	3.9 ± 1.8	4.3 ± 1.6	0.042
Postop 1 month	3.6 ± 1.1	4.3 ± 1.7	0.039
Postop 3 months	3.3 ± 1.2	4.1 ± 1.1	0.041
Postop 6 months	2.8 ± 1.3	3.6 ± 1.4	0.032
Postop 12 months	2.3 ± 1.4	2.6 ± 1.2	0.047
Postop 24 months	2.1 ± 1.1	2.2 ± 1.4	0.029
ROM			
preop	95.1 ± 7.7	96.2 ± 7.9	0.713
Postop 1 week	102.6 ± 9.8	97.9 ± 8.9	0.036
Postop 1 month	105.6 ± 7.2	98.9 ± 7.8	0.032
Postop 3 months	107.8 ± 7.3	100.9 ± 5.4	0.043
Postop 6 months	108.1 ± 8.2	104.3 ± 9.8	0.046
Postop 12 months	110.6 ± 7.1	109.9 ± 5.8	0.021
Postop 24 months	115.2 ± 8.2	114.9 ± 6.8	0.037

*VAS* Visual analogue score for pain; *ROM* Range of motion; *Preop* Preoperation; *Postop* Postoperation; mean ± standard deviation

**Table 4:** The KSS scores in the two groups

Results	Group S	Group E	*p*-value
Clinical score			
preop	36.4 ± 5.2	36.1 ± 4.6	0.781
Postop 1 week	52.1 ± 8.8	47.3 ± 7.2	0.041
Postop 1 month	72.4 ± 6.7	67.6 ± 5.9	0.038
Postop 3 months	79.6 ± 6.9	74.3 ± 4.7	0.031
Postop 6 months	82.4 ± 5.7	78.6 ± 5.9	0.041
Postop 12 months	88.1 ± 3.7	87.6 ± 3.1	0.046
Postop 24 months	92.5 ± 4.7	91.3 ± 4.9	0.039
Functional score			
preop	37.1 ± 5.4	38.6 ± 3.9	0.713
Postop 1 week	48.6 ± 8.3	44.1 ± 7.6	0.031
Postop 1 month	65.2 ± 6.8	61.1 ± 5.7	0.026
Postop 3 months	71.1 ± 5.8	67.4 ± 4.2	0.035
Postop 6 months	76.4 ± 4.2	72.1 ± 3.8	0.046
Postop 12 months	81.1 ± 3.8	80.2 ± 3.2	0.025
Postop 24 months	85.6 ± 3.6	84.1 ± 3.4	0.044

*KSS* Knee Society Score; *Preop* Preoperation; *Postop* Postoperation; mean ± standard deviation

**Table 5:** The FJS scores in the two groups

Results	Group S	Group E	*p*-value
Postop 1 week	44.9 ± 8.3	40.2 ± 8.6	0.046
Postop 1 month	57.4 ± 5.7	50.6 ± 8.9	0.038
Postop 3 months	62.6 ± 5.1	55.4 ± 7.5	0.026
Postop 6 months	71.3 ± 4.6	65.6 ± 5.3	0.035
Postop 12 months	78.4 ± 3.7	70.6 ± 6.9	0.039
Postop 24 months	81.2 ± 3.4	78.1 ± 4.7	0.041

*FJS* Forgotten Joint Score; *Preop* Preoperation; *Postop* Postoperation; mean ± standard deviation

**Table 6:** Multiple linear regression analysis

	Coefficient	95% CI	*p-*value
Scalpel method	39.8	26.2 to 74.1	0.029
Electric cautery method	29.1	22.6 to 61.3	0.157
Age	0.735	0.216 to 1.314	0.652
BMI	−0.684	−1.227 to − 0.517	0.071
Sex	0.618	−1.318 to 3.262	0.738

*BMI* Body mass index; *CI* Confidence interval

## Discussion

The most significant finding in this research was that the patients who underwent TKA by using the scalpel could achieve better clinical outcomes. In addition, if forgotten artificial joint after TKA was the final goal, the patients who underwent TKA by using the scalpel would acquire better quality of life.

Blood loss is an major problem in the process of TKA, which will cause many related complications. TXA has been suggested as a treatment option to reduce blood loss during TKA. Some studies have indicated that the intravenous use of TXA in the perioperative period has achieved significant effects in reducing blood loss [18, 19]. Similarly, studies have confirmed that topical use of TXA can obtain similar clinical effects as intravenous use of TXA, and the less possibility of complications [20, 21]. In addition, other studies have pointed out that the topical use of the hemostatic agent, such as Floseal®, has achieved significant results in controlling blood loss during TKA [22–25]. In the present study, in order to reduce the related complications caused by blood loss, all of our patients have combined intravenous and topical use of TXA. Most surgeons believed that, like abdominal surgery [26] and spinal surgery [27], electric cautery could reduce blood loss during TKA. In present study, we did not calculate the estimated blood loss by weighing the gauze and calculating the amount of liquid in the suction bottle. Instead, we adopted a method mentioned in the previous article [28], the blood loss was compared between the two groups by comparing haemoglobin, hematocrit before and after surgery. It has known that one unit of blood loss has an effect of 3% on hematocrit levels and 1 g/dL on hemoglobin. In our research, we found that there was no significant difference in the hemoglobin levels and hematocrit whether using the electric cautery or the scalpel. Perhaps it was related to the tourniquet, tranexamic acid, and hypotensive anesthesia with low mean arterial pressures during surgery. Furthermore, Tammachote et al. [11] believed that the main cause of intraoperative blood loss might be due to osteotomy and femoral medullary hemorrhage during surgery.

Postoperative wound infection is one of the most severe complications after TKA. The causes of infections are diverse, for example, incision hematoma, contaminated incisions, and less stringent aseptic procedures. Although we used broad-spectrum antibiotics to prevent this problem, we had not got satisfactory results. Some studies have pointed out [29–31] that electric cautery may cause delayed wound healing and histological indicate that tissue damage from electric cautery can easily cause tissue damage and increase the infection rate of the incision site. In addition, previous researches have reported that the inflammatory response at the wound affects early functional exercise after joint replacement [32, 33]. And, the damage caused by electric cautery to surrounding tissues may be the main factor leading to the inflammatory response [13, 14]. These findings were consistent with our study that higher wound complications were found by using the electric cautery after TKA. In order to prevent surgical site infection and wound complications, the Centers of Disease Control and Prevention has put forward prevention guidelines [34]. In addition, as an alternative to traditional dressings, negative-pressure wound therapy (NPWT) has been used to effectively treat open wounds in various situations [35, 36]. Recently, there is increasing evidence that closed incisional NPWT (ciNPWT) can potentially reduce the risk of surgical site infection, wound complications, reoperation, and decreased length of hospitalization in patients with TKA [37–39]. Although we did not use this kind of ciNPWT in our study, it might be a better choice when we encounter severe wound complications in the future.

The FJS is a newly developed scoring system in recent years, which is often used to measure patients’ ability to forget joint replacement or joint awareness in daily life. Even if the patient’s knee function is improved and no pain is felt, the FJS score will be lower if the patient is “aware of” the presence of artificial joints in daily life. As a result, minor complaints that are not identified by specific issues (such as “Can you do sports?”) are called “aware” joints, which may reduce the ceiling effect and more sensitively reflect postoperative quality of life [17, 40]. Ozaki et al. believed that FJS is a scoring system that can express “sense of stability” as “awareness” [41]. Morten et al. believed that FJS combines factors such as stiffness, pain, the ability of daily activities, and patients’ expectations to reflect patients’ ability to forget artificial joints in activities, so this scoring system may be the best tool to evaluate the results of TKA [42]. Another study found that when using the FJS scoring system to evaluate the difference in knee awareness of patients who underwent patellofemoral arthroplasty, unicompartmental knee arthroplasty and TKA treatment, they found that patients who underwent different joint arthroplasties had very large differences in the FJS [43]. In the present study, we found higher FJS score by using the scalpel after TKA, and this might mean that the patients had a higher quality of life.

Increased potential smoke plume risk is another risk factor of electric cautery [11, 12]. Surgical smoke exposure may increase the risk of acute or chronic lung diseases such as pneumonia or asthma. One study noted that in Mexico, as a result of exposure to electric cautery smoke, many surgical surgeries developed lump (58%) and sore throat (22%) in the throat [44]. Some studies have also shown that perioperative nurses have twice as many respiratory diseases as asthma, bronchitis, allergies, and sinus infections in the general population [45, 46]. Therefore, for the safety of patients and medical staff, we should pay more attention to the problem of smoke generated by electric cautery during surgery. However, a previous study pointed out that traditional surgeries with the scalpel were more cost-effective than the electric cautery [11]. Therefore, we recommended that using the ordinary scalpel as much as possible when performing primary TKA.

The limitation of this research was that it had a retrospective short-term follow-up design, which has its potential weaknesses. A prospective and long-term research should be performed to confirm these findings.

## Conclusion

In this research, the patients who underwent TKA by using the scalpel could achieve better clinical outcomes. In addition, if forgotten artificial joint after TKA was the final goal, the patients who underwent TKA by using the scalpel would acquire better quality of life.

## Data Availability

The detailed data and materials of this study are available from the corresponding author via e-mail on reasonable request.

## Abbreviations

TKA: Total knee arthroplasty
VAS: Visual analog score
ROM: Range of motion
KSS: Knee society score
FJS: Forgotten joint score
OA: Osteoarthritis
BMI: Body mass index
TXA: Tranexamic acid
